# Identifying patients with asthma-chronic obstructive pulmonary disease overlap syndrome using latent class analysis of electronic health record data: a study protocol

**DOI:** 10.1038/s41533-018-0088-4

**Published:** 2018-06-20

**Authors:** Mohammad A Al Sallakh, Sarah E Rodgers, Ronan A Lyons, Aziz Sheikh, Gwyneth A Davies

**Affiliations:** 1 Swansea University Medical School, Singleton Park, Swansea, SA2 8PP UK; 2 Asthma UK Centre for Applied Research, Edinburgh and Swansea, UK; 3 The Farr Institute of Health Informatics Research, Edinburgh and Swansea, UK; 4 Usher Institute of Population Health Sciences and Informatics, The University of Edinburgh, Edinburgh, UK

## Abstract

Asthma and chronic obstructive pulmonary disease (COPD) are two common different clinical diagnoses with overlapping clinical features. Both conditions have been increasingly studied using electronic health records (EHR). Asthma-COPD overlap syndrome (ACOS) is an emerging concept where clinical features from both conditions co-exist, and for which, however, there is no consensus definition. Nonetheless, we expect EHR data of people with ACOS to be systematically different from those with “asthma only” or “COPD only”. We aim to develop a latent class model to understand the overlap between asthma and COPD in EHR data. From the Secure Anonymised Information Linkage (SAIL) databank, we will use routinely collected primary care data recorded in or before 2014 in Wales for people who aged 40 years or more on 1st Jan 2014. Based on this latent class model, we will train a classification algorithm and compare its performance with commonly used objective and self-reported case definitions for asthma and COPD. The resulting classification algorithm is intended to be used to identify people with ACOS, ‘asthma only’, and ‘COPD only’ in primary care datasets.

## Background

Asthma and chronic obstructive pulmonary disease (COPD) are two common different clinical diagnoses with overlapping clinical features. Global Initiative for Asthma (GINA) defined asthma based on variable respiratory symptoms and expiratory airflow limitation.^1^ On the other hand, the Global Initiative for Chronic Obstructive Lung Disease (GOLD) defined COPD based on persistent respiratory symptoms and airflow limitation.^2^ While asthma affects people from the early school age, COPD mainly affects those aged over 40 years with a smoking history. Clinically, the differentiation between the two diseases and identifying their overlap in those older people can be challenging.^1^ Co-existence of clinical features of both conditions along with persistent airflow limitation has been recently recognised by a joint committee publication between GOLD and GINA as the asthma–COPD overlap syndrome (ACOS).^3^

However, there are currently no universally agreed consensus clinical definitions for the diagnosis of asthma,^4–9^ COPD,^10,11^ and ACOS.^12–15^ Subsequently, the prevalence of these three conditions is highly dependent on case definitions and data sources.^16–20^

In studies conducted using electronic health records (EHR), identifying patient groups is further complicated by the limitations of these data, such as missing data and coding errors.^21–23^ Despite the lack of consensus clinical definitions, we expect EHR data of people with ‘ACOS’ to be systematically different from those with ‘asthma only’ or ‘COPD only’. Case definitions aiming to differentiate between those patient groups based solely on clinical knowledge or face validity may be inaccurate, and validating them with traditional methods, e.g., review of full patient records, is time consuming and labour intensive. Clustering methods overcome these challenges by automatically identifying subgroups in the population that best explains the patterns in high-dimensional EHR data, without an a priori hypothesis about those subgroups and their labels.^24^ Latent class analysis (LCA) is such a method that can probabilistically identify patients with asthma and/or COPD using the available recorded data.

## Aims

We plan to develop an LCA model to identify and characterise patients with asthma, COPD and ACOS in Wales. Based on this LCA model, we will derive a classification algorithm and compare its performance with commonly used objective and self-reported case definitions for asthma and COPD.

## Methods

We will use primary care data on asthma and COPD recorded in or before 2014 for a sample of the Welsh population to find, using LCA, clinically meaningful classes (i.e., clusters) related to the two conditions in that year. We will follow the STROBE^25^ and RECORD Statements^26^ in reporting the full study.

### Data sources

We will use the following two deidentified datasets from the Secure Anonymised Information Linkage (SAIL) Databank in Wales:^27,28^

- The Welsh Demographic Service (WDS) which contains demographic and administrative information for the National Health Services (NHS) patients in Wales.
- The General Practitioner (GP) dataset which contains primary care events, such as diagnoses, clinical findings, and prescriptions codified in Read codes by general practitioners.

At the time of writing of this protocol, the most recent extract of the GP dataset was in March 2017, covering about 80% of GP surgeries in Wales.

### Patient population

The study sample will be randomly selected from the total population of Wales within the SAIL Databank in 2014. The sampling will be stratified by general practices to improve their representativeness. We will determine the sample size based on the computational capacity in the SAIL Databank which will be available for this study. The sampling frame will include all individuals who were aged at least 40 years on 1st January 2014.

### Latent class modelling

LCA is a finite mixture modelling method that aims to divide a sample into classes or clusters related to a set of observed variables.^24,29^ LCA assumes that the patterns in these observed variables can be explained by, in addition to measurement errors, a hidden categorical variable that divides the sample into a pre-defined number of distinct classes.

In our study, we will construct observed variables from asthma- and COPD-related events recorded in the GP Dataset. The construction of observed variables will be based on their usefulness, from a clinical perspective, for identifying and distinguishing between patients with asthma and/or COPD. These variables will include diagnosis, GP visits, and prescriptions related to asthma and COPD, as well as history of allergy (including atopic eczema/dermatitis, food allergy, allergic rhinitis, and anaphylaxis) and smoking history (see Table 1). GP visits and prescriptions will be queried during 2014, while the other events will be queried in or any time before 2014.

**Table 1:** Observed variables that will be used in the latent class model

Variable	Time interval for calculation	Categories
Asthma related		
Asthma diagnosis codes	Ever	0, 1+
Age at asthma first diagnosis codes (if any)	–	<40, ≥40, no diagnosis
Asthma GP visits codes	Last year	0, 1+
COPD related		
COPD diagnosis codes	Ever	0, 1+
COPD GP visits codes	Last year	0, 1+
COPD-specific prescriptions codes^*^	Last year	0, 1+
Prescriptions		
ICS codes	Last year	0, 1+
SABA codes	Last year	0, 1+
LABA codes	Last year	0, 1+
ICS+LABA codes	Last year	0, 1+
OCS codes	Last year	0, 1+
LTRA codes	Last year	0, 1+
Others		
Allergy history^**^	Ever	No, yes
Smoking history	Ever	No, yes
Gender	–	Male, female

Abbreviations: *COPD* = chronic obstructive pulmonary disease, *ICS* = inhaled corticosteroids, *GP* = general practitioner, *LTRA* = leukotriene receptor antagonists, *LABA* = long-acting *β*2 agonists, *OCS* = oral corticosteroids, *SABA* = short-acting *β*2 agonists.

^*^COPD-specific prescriptions include: glycopyrronium bromide, indacaterol, olodaterol, anticholinergic bronchodilators (ipratropium bromide, oxitropium bromide, tiotropium, aclidinium, umeclidinium), roflumilast, oxygen cylinders, and COPD rescue packs.

^**^Allergy includes atopic eczema/dermatitis, food allergy, allergic rhinitis, and anaphylaxis.

Model parameters will include proportions of the latent classes and probabilities of observing the levels of observed variables in each latent class, a.k.a item–response probabilities. Parameters will be estimated by the expectation–maximisation (EM) algorithm, which iteratively searches for maximum–likelihood parameter values for which the data are more likely to be observed.^30^ Based on observed characteristics, each individual is assigned membership probability in each latent class^29^ and is finally assigned to the latent class of maximum membership probability.^31^

We will begin the modelling for two latent classes and will then iteratively increase the numbers of latent classes. Model selection will be based on model diagnostics and interpretability.

We will look for a model for which the Bayesian Information Criterion (BIC)^32,33^ is ideally minimum, or becomes ‘stabilised’, indicating no significant improvement in information gain beyond a certain number of classes. In addition, the selected model should be clinically relevant; we will use the estimated item–response probabilities to assign labels consistent with ‘asthma’, ‘COPD’, ‘both’ (ACOS), and ‘none’ to the latent classes. We will use class shares as prevalence estimates for these clinical labels among the age groups of 40 and over in 2014.

LCA modelling will be performed using the R package *poLCA* (version 1.4.1, 2014).^34^

### Derivation of a classification algorithm

Based on the LCA model, we will derive a classification algorithm to identify patients with asthma, COPD and ACOS according to their characteristics. To do so, we will perform recursive partitioning^35^ using the assigned latent classes as labels and the aforementioned observed variables as predictors. We will use the R package *rpart* (version 4.1–11, 2017)^36^ for this purpose.

### Comparison with other case definitions

We will compare the LCA model and the derived classification algorithm with other objective and self-reported measures. As objective measures, we will use definitions used in the Quality of Outcomes Framework (QOF) 2014–2015 indicators for ‘treated asthma’ (AST001) and ‘COPD’ (COPD001).^37^ From the Welsh Health Survey (WHS) 2014,^38^ we will use self-reported responses on current treatment of ‘asthma’, ‘emphysema’, and ‘spells of bronchitis that have lasted over 3 years’, with any of the latter two representing currently-treated COPD. We will treat invalid and missing responses as negative responses. We will perform the comparisons in the group of the WHS 2014 participants who were aged 40 years or over on 1st January 2014, and whose responses where successfully linked to the SAIL Databank. We will calculate diagnostic accuracy measures of the LCA model and the classification algorithm against each of the above case definitions and vice versa.

### Ethics, timeline and dissemination

We obtained an approval to use the SAIL Databank from the Information Governance Review Panel. NHS Research Ethics Committee approval for this study is not required because we will only use anonymised data. The data extraction and statistical analysis will be performed between March and May 2018. The full paper will be submitted for publication in a respiratory care-related peer-reviewed journal in due course.

## Discussion

While the interest in ACOS is growing, there is no consensus definition for this emerging and debated concept,^39^ leading to wide variations in prevalence and impaired comparability between studies. With the increasing use of EHR data to study asthma and COPD, it is important to develop operational definitions for ACOS based on such data. In this study, we will perform LCA on recorded events of diagnosis, prescriptions, and healthcare utilisation for asthma and COPD in routinely collected primary care data. By including observed variables for asthma and COPD in the same model, we will be able to identify patients with either or both conditions (i.e., ACOS).

An inherent limitation of routinely collected EHR data is the lack of vital pieces of information that are often used to make diagnoses at the point of care. Unlike diagnosis and prescriptions which are generally well coded, important diagnostic tests such as lung function and peripheral eosinophil count are often poorly and inconsistently recorded in primary care datasets. These missing data would have been potentially useful for improving the accuracy of our model. However, it is often difficult to assess data missingness in event-based databases. The GP Dataset in the SAIL Databank is a long-format dataset, in which each row contains a dated code representing a single primary care event. The presence of a code usually indicates that the corresponding event occurred. However, when a code is absent, it is often impossible to ascertain whether the event did not occur or whether it was simply not recorded or coded. This is a particular challenge for events that are known to be poorly recorded. Therefore, since the quality of observed variables is essential in LCA, we will only include variables that are thought to be of reasonable quality in the SAIL Databank. In interpreting the results, we will consider the limitations of EHR-derived data such as the possibility of missing or incorrect codes and the changes in coding practices over time.

LCA itself has limitations. The construction of observed variables, model selection and interpretation involves a level of subjectivity. The model’s interpretation and usefulness depends largely on the choice and structure of observed variables. In our LCA modelling, the clinical meaning of the latent classes will be based on surrogate variables, such as diagnosis, GP visits, and prescriptions, rather than on more direct disease markers such as clinical and laboratory findings. Nevertheless, we hypothesise that LCA of these surrogate variables can reasonably distinguish between patients with asthma, COPD, and ACOS. This will also provide an opportunity to assess how clustering based on these surrogate variables will perform compared with that based on disease markers.^40–47^ Comparing the LCA model and the classification algorithm against other objective and self-reported measures will provide useful information about their validity and performance.
